# We Must Abandon the Myth: Oxidized Low-density Lipoprotein is not a Lipoprotein that Plays a Key Role in Atherogenesis

**DOI:** 10.2174/0109298673301236240311113807

**Published:** 2024-03-15

**Authors:** Alexander N. Orekhov

**Affiliations:** 1 Institute of General Pathology and Pathophysiology, Moscow, Russia

**Keywords:** Atherogenesis, autoantibodies against LDL, desialylated LDL, electronegative LDL, malondialdehyde treated LDL, multiple-modified LDL, oxidized LDL, small dense LDL

## Abstract

The purpose of this review is to revisit in detail the arguments supporting or disproving the hypothesis that oxidized low-density lipoprotein (LDL) plays a key role in atherosclerotic lesion development. The detection of oxidized LDL *in vivo* was extremely important for confirming its key role in atherogenesis. Indirect evidence of its existence included the presence of autoantibodies against malondialdehyde-treated LDL in human blood; however, the affinity of circulating antibodies to another LDL modification, such as desialylated LDL, was an order of magnitude stronger. At least 3 forms of atherogenic modified lipoproteins were isolated from the blood of atherosclerotic patients using different methods, namely, small dense, electronegative and desialylated. Their properties were so similar that it was suggested that the three types could be classified as the same multiple-modified LDL particle. It has been shown that when native (unmodified) LDL is incubated with autologous serum from patients with atherosclerosis, multiple modifications occur, which include desialylation, a decrease in the content of phospholipids and neutral lipids, a decrease in particle size, an increase in negative charge and other physical and chemical changes. Longer incubation also increased the susceptibility of LDL to oxidation. Thus, LDL oxidation is not the only, much less the most important, form of atherogenic modification of LDL since it occurs at the last stages of multiple modifications cascade and does not significantly increase the atherogenic potential of multiple-modified LDL. Finally, clinical trials did not support the oxidative hypothesis; however, research on oxidized LDL continues, influencing the future research. It is time to abandon the myth.

## 1 INTRODUCTION

The hypothesis that the oxidized form of low-density lipoprotein (LDL) plays a key role in atherogenesis was widely disseminated by Dr. Dan Steinberg about 40 years ago [1]. The idea was quickly picked up, and over the next 20 years, the number of articles mentioning oxidized LDL in relation to atherosclerosis grew exponentially, reaching a plateau with a maximum of approximately 300-350 per year. In total, more than 12 thousand articles with keyword “oxidized LDL” has been published. Such popular adoption made the oxidized LDL hypothesis paradigm-forming, largely influencing the lines of further research. Although other (non-oxidative) forms of modified LDL have been found in the blood of patients with atherosclerosis [2-4], oxidized LDL is still largely considered the only atherogenic modified lipoprotein [5-7]. It is, however, useful to revisit our understanding of the role of modified LDL forms in atherogenesis, taking into consideration other forms than oxidized LDL. Accumulated evidence demonstrated that oxidized LDL was not an optimal model for atherogenesis studies *in vivo* and *in vitro*, and its presence in circulating blood turned out to be difficult to prove [8-10]. More recent data accumulated on the topic fails to support the oxidized LDL hypothesis and highlights the need for more accurate studies of atherogenic LDL modification. The purpose of this report is to review in detail the arguments for and against the oxidized LDL hypothesis.

## 2 LDL AND ATHEROSCLEROSIS

**Fig. (1):**
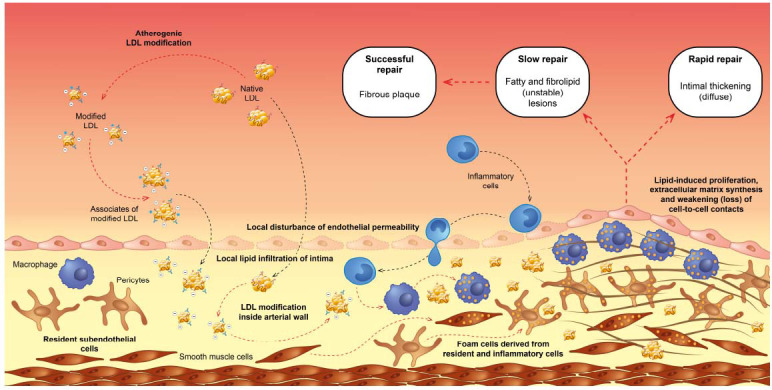
Mechanisms of participation of modified LDL in atherogenesis.

The extra- and intracellular deposition of lipids, especially cholesteryl esters, within the vascular intima (the innermost layer of the arterial wall) is one of the earliest manifestations of atherosclerosis [11]. This lipid accumulation in the arterial wall is a key feature of the disease (Fig. **1**). In the early stages of atherosclerosis, circulating LDL particles penetrate to the arterial intima, where they are taken up by cells populating the arterial wall, leading to the formation of foam cells [12]. Foam cells are lipid-laden macrophages, macrovascular pericytes and smooth muscle cells that are considered a hallmark of early atherosclerotic lesions [13-16]. The lipids accumulated in the arterial intima are primarily cholesteryl esters, which are formed by the esterification of cholesterol with fatty acids and represent the major cellular storage form of cholesterol. As foam cells continue to accumulate lipids, the deposition of cholesteryl esters contributes to the formation of fatty streaks, which are the earliest visible lesions in the development of atherosclerosis. The deposition of cholesteryl esters and other lipids in the arterial intima triggers a cascade of events, including inflammation, recruitment of immune cells, smooth muscle cell migration, and the formation of fibrous plaques [17-22]. Over the time, these processes can lead to the narrowing of arteries, impaired blood flow, and the potential for plaque rupture, which can trigger acute cardiovascular events such as heart attacks or strokes. Therefore, the early extra- and intracellular deposition of cholesteryl esters within the vascular intima plays a crucial role in the initiation and progression of atherosclerosis, making it an important target for understanding and managing the disease.

Studies of atherosclerosis models have shown that native LDL alone is generally not effective in inducing significant intracellular accumulation of cholesteryl esters. Native LDL particles, in their unmodified form, are typically not efficiently taken up by cells in a way that leads to substantial cholesteryl ester accumulation [23]. The primary reason for this is that native LDL is recognized by specific cell surface receptors, such as the LDL receptor, which facilitate the cellular uptake of LDL through receptor-mediated endocytosis [23, 24]. This internalization pathway is tightly controlled, and the conversion of cholesterol delivered by this pathway into cholesteryl esters and subsequent accumulation is limited.

On the other hand, LDL chemically modified *in vitro* (acetylated, malondialdehyde-treated, oxidized with ions of transient metals, *etc.*) induced lipid deposition in cells, *i.e.*, was shown to be atherogenic [25-29]. Acetylated LDL, resulting from the acetylation of specific lysine residues on the apolipoprotein B-100 (apoB-100) protein of LDL, has been extensively studied in the context of atherosclerosis [30-32]. Acetylated LDL is taken up by macrophages through unspecific scavenger receptors, leading to the formation of foam cells [33, 34]. Malondialdehyde (MDA) is a reactive aldehyde produced during lipid peroxidation processes [35]. MDA-modified LDL particles are also recognized by scavenger receptors on macrophages, leading to foam cell formation and the initiation of atherosclerosis [36, 37]. Oxidized LDL is another form of chemically modified LDL that is generated by various oxidative modifications, including exposure to reactive oxygen species, transition metal ions, or enzymes. Oxidized LDL is taken up by macrophages through scavenger receptors, contributing to the formation of foam cells and the progression of atherosclerosis [38, 39]. These chemically modified forms of LDL induce various cellular responses, including upregulation of adhesion molecules, production of inflammatory cytokines, and the formation of foam cells [40-43]. However, attempts to isolate modified types of LDL from human blood corresponding to the oxidized forms studied *in vitro* have proven difficult and unsuccessful.

During the early 1980s, a paradoxical situation was in place regarding LDL and its role in atherosclerosis. It was known that LDL can contribute to the accumulation of lipids in the arterial walls, which is a hallmark of atherosclerosis [44]. This understanding was supported by evidence from various studies, including *in vitro* experiments and animal models. Modified forms of LDL can undergo cellular uptake by macrophages, leading to the formation of foam cells [45]. However, native or unmodified LDL does not typically induce the accumulation of intracellular fat or foam cell formation [46]. These particles are recognized by LDL receptors on the surface of cells, allowing for the regulated uptake of cholesterol for cellular needs [47]. *In vitro*-modified LDLs were known to be atherogenic, but they could not be isolated from the blood of patients. This paradox in the early 1980s stemmed from the understanding that LDL, when modified, can contribute to atherosclerosis by promoting lipid accumulation in the arterial wall, and this prompted an intense search for modified *in vivo* LDL capable of inducing intracellular lipid accumulation.

## 3 MECHANISMS OF PARTICIPATION OF MODIFIED LDL IN ATHEROGENESIS

The importance of searching for atherogenic *in vivo* modified LDL in human blood was key to understanding the mechanisms of initiation and development of atherosclerotic lesions. These mechanisms are summarized in Fig. (**1**).

Atherogenic modification of LDL occurs both in the blood and in the vascular wall. A cascade of multiple modifications can occur in the blood, which is described in detail below (section 7, oxidation does not contribute significantly to the atherogenicity of LDL). Modified LDL, including oxidized LDL, was detected in the arterial intima and studied in depth [48, 49].

Modified LDL has a tendency to self-associate and also binds to autoantibodies, forming circulating immune complexes [50, 51]. If such formations are not removed from the bloodstream, then they penetrate into the arterial wall, where they cause a much greater accumulation of intracellular lipids compared to single dispersed LDL particles [52].

Modified LDL, including as part of associates or complexes, accumulates in the subendothelial intima locally due to local disturbances in endothelial permeability [53]. This explains the fact that atherosclerosis affects the arterial wall not diffusely but mosaically. LDL associates or LDL containing complexes will be taken up by subendothelial cells (macrophages, pericytes and smooth muscle cells) by phagocytosis, bypassing the specific receptor-mediated pathway of intracellular metabolism of the lipoprotein particle [54].

Phagocytosis of large lipoprotein formations is a trigger for the innate immune response [10]. In particular, this response is manifested in the recruitment of immune cells to the area of potential inflammation. Monocytes that enter the arterial wall differentiate into macrophages and begin to phagocytose large lipoprotein formations, enhancing the innate immune response.

Since the modified LDL enters the cell bypassing the natural specific metabolic pathway, excess lipid accumulates in the cell in the form of lipid granules, droplets and larger ovoid formations, which fill the entire cytoplasm, creating a foamy appearance for the cell. Foam cells are the earliest and most visible manifestation of atherosclerosis at the cellular level. The accumulation of intracellular lipids causes an increase in the proliferative activity of intimal cells as well as the synthesis of the extracellular matrix and promotes further LDL uptake and lipid accumulation [55]. Excess fat in cells provokes a weakening of intercellular communications and even loss of cell-to-cell contacts [56].

With a favorable development of events, the innate immune response (acute inflammatory reaction) quite quickly ends in resolution. At the site of local inflammation, a slight compaction and some thickening of the intima remains. Throughout life, local inflammation occurs in the arterial wall here and there; therefore, with age, the intima thickens diffusely, and this is called diffuse intimal thickening, which is not an atherosclerotic lesion. Local disruption of the mechanisms of innate immunity can lead to the chronification of inflammation [57] that can last for years and decades, accompanying the development of atherosclerotic lesions and creating a threat to health and life. The successful completion of this process can be considered the formation of a powerful fibrous cap over the focus of inflammation, which separates the focus from the lumen of the vessel and prevents further penetration of modified LDL into this zone. The lesion stabilizes in the form of a fibrous plaque that is not life-threatening.

## 4 THE APPEARANCE OF OXIDIZED LDL HYPOTHESIS

The idea of chemical modification of LDL as a mechanism for atherogenesis has been widely promoted by Dr. Joseph L. Witztum and Dr. Daniel Steinberg since the early 1980s. In their works, published in 1984, Witztum and Steinberg suggested that oxidative modification of LDL particles played a crucial role in the development of atherosclerosis [25]. They proposed that LDL, when exposed to oxidative stress in the arterial wall, undergoes various chemical modifications, such as oxidation of its lipid components and structural alterations of its protein moiety. These modified LDL particles were hypothesized to become atherogenic by triggering a series of inflammatory and cellular responses in the arterial wall, leading to the formation of fatty plaques and the progression of atherosclerosis. This concept, known as the “oxidized LDL hypothesis,” provided a new perspective on the role of LDL in cardiovascular disease. Since its proposal, extensive research has been conducted to explore the mechanisms and consequences of LDL modification, confirming its importance in atherosclerosis development and highlighting its potential as a therapeutic target for preventing and treating cardiovascular diseases.

Of note, Steinberg's team did not demonstrate intracellular lipid accumulation caused by oxidized LDL, although this was the cornerstone of the evidence for the atherogenicity of the oxidative form of lipoprotein particle modification. This was shown by other authors in 1984, who found that *in vitro* incubation of mouse peritoneal macrophages with oxidized LDL resulted in a 6- fold increase in cholesterol ester, while incubation with native LDL did not result in cholesterol accumulation [58]. Steinberg *et al.* never cited this work. On the other hand, they referred to a 1980 paper that showed cholesteryl ester accumulations in human monocyte-macrophages incubated with MDA-LDL [59]. However, it must be admitted that such a reference was not entirely correct.

## 5 ATTEMPTS TO FIND OXIDIZED LDL IN THE BLOOD

As stated above, all attempts to find the species of modified LDL known from the *in vitro* experiments in circulation have been difficult and unsuccessful. The detection of oxidized LDL *in vivo* was extremely important for building a system of evidence for its key role in atherogenesis. A 1989 paper by Steinberg's group categorically stated that evidence for the existence of oxidized LDL *in vivo* had been found. The title of the article, “Low-density lipoprotein undergoes oxidative modification *in vivo*” speaks for itself [60]. The paper presented “three mutually supportive lines of evidence for oxidation of LDL *in vivo*: (i) antibodies recognizing oxidized LDL, malondialdehyde-lysine, or 4-hydroxynonenal-lysine recognize materials in the atherosclerotic lesions of LDL receptor-deficient rabbits; (ii) LDL gently extracted from lesions of these rabbits was recognized by an antiserum against malondialdehyde-conjugated LDL; (iii) autoantibodies against malondialdehyde-LDL (titers from 512 to greater than 4096) could be demonstrated in rabbit and human sera.” However, circulating oxidized LDL itself has not been isolated from human blood and has therefore not been characterized. Thus, the strongest evidence relied on autoantibodies against MDA-LDL.

However, another group soon published evidence that circulating anti-LDL autoantibodies have an affinity not only for MDA-LDL but also for other forms of lipoprotein particles [61]. In particular, the affinity of anti-LDL to LDL desialylated *in vitro* with neuraminidase was an order of magnitude higher than that of MDA-LDL (compare 89.4 *vs.* 10.9 × 10-7M-1). This has only one explanation, namely: the conformation of the circulating modified LDL is such that there is an epitope with a very high affinity for desialylated LDL and a lower affinity for MDA-LDL. Most likely not MDA-LDL, but desialylated LDL found in the blood of atherosclerotic patients in 1989 [62] was a natural anti-LDL antigen. It follows that Steinberg's group did not prove that LDL oxidation occurs *in vivo*, since the detection of antibodies to MDA-LDL cannot be considered as solid proof. On the other hand, other forms of atherogenic modification, including desialylated LDL, circulate in the blood and can be isolated and characterized.

## 6 REAL (NOT MYTHICAL) FORMS OF ATHEROGENIC-MODIFIED LDL CIRCULATING IN THE BLOOD

In 1988, a circulating subclass of LDL was described as “small dense LDL” (sdLDL) [62]. Further studies by Krauss *et al.* convincingly demonstrated the close association of this LDL subclass with atherosclerosis and atherosclerotic diseases [63-66].

In 1988, electronegative LDL circulating in the blood was described [67]. This form of atherogenic modification of LDL was isolated and characterized [68-70]. Electronegative LDL has been widely studied so far in both basic and translational studies [71-80].

As mentioned above, in 1989, desialylated LDL was found in the blood of atherosclerotic patients and was identified as having atherogenic properties; that is, it could cause the accumulation of intracellular lipids in cell culture [61]. A joint study of two scientific groups showed that the more electronegative LDL isolated by ion-exchange chromatography was desialylated LDL [81].

It has been independently found that the subfraction of desialylated LDL was more electronegative [82]. Both facts suggested that desialylated LDL and electronegative LDL represent the same lipoprotein particle with a reduced content of sialic acid and an increased negative charge.

The desialylated LDL particle was found to be smaller and denser than the native LDL particle [82]. Independently, La Belle and Krauss have shown that sdLDL had a low sialic acid content, *i.e.*, sdLDL is desialylated [83]. Thus, desialylated and sdLDL could also represent the same particle. Table **1** lists changes in physical, chemical and other characteristics of three forms of atherogenic modified LDL detected in human blood as compared to native LDL, demonstrating similarity between these characteristics. For instance, the sialic acid content in all three forms is lower than in native LDL, while the susceptibility to oxidation is higher. This suggests that all subfractions of atherogenic modified LDL isolated and detected by different methods could be the same lipoprotein particle subjected to multiple modifications. It should be noted that modified LDL possesses an elevated susceptibility to oxidation, probably due to reduced levels of antioxidants (Table **1**). However, judging by the above, oxidation cannot be the main cause of LDL modification.

## 7 OXIDATION DOES NOT CONTRIBUTE SIGNIFICANTLY TO THE ATHEROGENICITY OF LDL

As discussed above, the available evidence leaves little doubt that natural atherogenic modification of LDL *in vivo* is a multiple modification. It is important to find out whether such a modification can occur in the blood. It was shown that after incubation for 24 h at 37°C in whole blood or plasma obtained from atherosclerotic patients, the sialic acid content of LDL was 2-fold lower than that of native LDL incubated with whole blood and plasma obtained from healthy individuals [98, 100]. Incubation with red and white blood cells had no effect on the sialic acid content. It can, therefore, be concluded that multiple modification of LDL takes place in blood plasma.

**Fig. (2):**
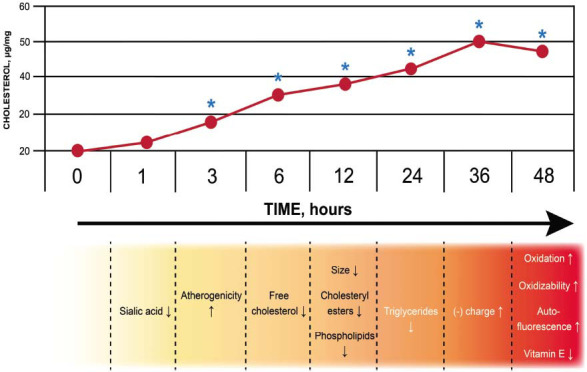
Cascade of multiple modification of LDL during incubation with serum. The top graph demonstrates the effect of LDL incubated with plasma-derived serum at 37°C on total cholesterol content (in μg/mg of cellular protein) in cells cultured from uninvolved intima of human aorta. Sialylated LDL was isolated by lectin chromatography and incubated with autologous plasma-derived serum. Cells were incubated with 100 mg/ml LDL for 24 h. Data are the means of three determinations ± S.E.M. *Significant difference from non-incubated LDL, *p* < 0.05. The bottom diagram shows the sequence of events occurring with native LDL in the plasma. “Already after 1 h of incubation the sialic acid content is decreased while the proportion of desialylated LDL increases. After 3 h of incubation LDL starts manifesting atherogenic properties. Within a 6-24-h interval, a fall in the levels of all major classes of lipids is observed, which in turn causes a reduction in the particle size. An increase in the electronegative charge is observed after 36 h of incubation while the first signs of lipid peroxidation and apo B degradation appear only after 48 h.”. Adapted from [100].

For a detailed study of the changes occurring in the LDL particle during incubation with the plasma of atherosclerotic patients, a special protocol was developed [100]. Native (normally sialylated) LDL was isolated from plasma by ultracentrifugation followed by lectin chromatography, which allowed the removal of desialylated particles. ApoB-containing lipoprotein-free serum was obtained, after which LDL and serum were mixed in proportion as in the original plasma and incubated at 37°C. After 1 hour of incubation, a decrease in the content of sialic acid in initially native (normally sialylated) LDL was observed (Fig. **2**).

As the level of sialic acid decreased, LDL became atherogenic, that is, capable of inducing the accumulation of total cholesterol in cells cultured from an unaffected human aortic intima. Already after 3 hours of incubation, the accumulation of intracellular cholesterol was statistically significant. After 6 hours of incubation, a decrease in the content of phospholipids and neutral lipids in the LDL particle was detected. In addition, the particle size decreased.

After 36 hours, an increase in the negative charge of the LDL particles was observed. Longer incubation of LDL with serum for 48 and 72 hours caused a loss of α-tocopherol, and, in parallel, an increase in the susceptibility of LDL to oxidation occurred. Simultaneously, degradation of apoB occurred.

Thus, desialylation of LDL particles is the very first or one of the first acts of modification and, apparently, is a sufficient prerequisite for the appearance of atherogenic properties in LDL. Subsequent modifications only increase the atherogenic potential of LDL.

Summarizing the above data, it can be argued that the multiple modification of LDL in plasma is a cascade of sequential step-by-step changes in the lipoprotein particle, starting with desialylation, followed by lipid loss, a decrease in particle size and an increase in its electronegative charge, as well as lipid peroxidation. This fully explains the detection in the blood of various forms of LDL modification, namely desialylated, small dense, and electronegative. It is probably in this sequence that LDL particles circulating in the blood are transformed. It is important to emphasize that LDL oxidation is not the only form of modification and may not be the most important modification since LDL oxidation occurs at the very last stages of multiple modifications and does not significantly increase the atherogenic potential of multiple modified LDL.

## 8 IS THERE OXIDIZED LDL IN THE BLOOD?

After the above facts, such a question seems quite natural. No one has been able to isolate oxidized LDL from human blood; however, theoretical considerations have been put forward about the presence of signs or markers of oxidation in LDL circulating in the blood of patients. Recently, the identification of oxidation markers (such as cholesteryl ester-OOH, triglyceride-OOH, free cholesterol-OOH, phospholipid- OOH, apoB100-MDA, and apoB100-DiTyr) in LDL isolated from the blood of patients and control subjects was reported [8]. Previously, evidence for the presence of oxidized LDL in the blood of patients was obtained and reported by Tertov *et al.* in 1995 [101]. The authors showed that LDL oxidation may lead to a formation of covalent links between lipids and apolipoprotein B. Protein-bound phospholipid residues were detected by nuclear magnetic resonance and colorimetric determination of phosphate. It has been demonstrated that the content of protein-bound sterols in multiple modified LDL of patients with coronary atherosclerosis is higher than that in native LDL. These results suggest that the level of protein-bound lipids may be a marker of LDL oxidation and can be used to evaluate the association between lipoprotein oxidation and atherogenesis. Thus, adducts of lipids, particularly cholesterol, with apolipoprotein B have been shown to be stable markers of human plasma LDL oxidation.

Later, an attempt was made by the same researchers to assess the relationship between the degree of plasma LDL oxidation, evaluated by the content of apoB-bound cholesterol and the ability of LDL to induce cholesterol accumulation in cultured human aortic intima pericyte-like cells, *i.e.*, LDL atherogenic potential [102, 103]. LDL samples of healthy subjects did not increase cholesterol content in cells cultured from grossly normal intima of the human aorta. Most LDL preparations isolated from coronary atherosclerosis patients with or without hypercholesterolemia stimulated intracellular cholesterol accumulation. However, the ability of human LDL to induce cholesterol accumulation in cells did not correlate with the degree of *in vivo* LDL oxidation. These results suggest that atherogenicity of LDL circulating in human plasma does not depend on the degree of lipid peroxidation in LDL particles.

It can be concluded that oxidized LDL actually circulates in the blood of patients with atherosclerosis. However, the atherogenicity of circulating LDL does not correlate in any way with the degree of its oxidation. It is likely that the proportion of oxidized LDL in the blood is too small compared to the proportion of non-oxidized forms of LDL for oxidized LDL to play a significant role in the formation of the atherogenic properties of the blood. Based on this assumption, it can be explained why oxidized LDL has not yet been isolated from the blood for further research. As shown above, oxidation can only be the terminal phase of the cascade of multiple modifications of lipoprotein particles in the blood; therefore, the proportion of hypothetical oxidized particles should be small. In addition, senescent particles are removed from the blood by liver cells, and the oldest oxidized particles will be removed first.

## 9 OXIDIZED LDL IS A POOR MODEL OF MODIFIED LIPOPROTEIN

Thus, naturally occurring multiply modified LDL circulates in the blood. Multiple modifications include chemical changes affecting protein, lipid and carbohydrate moiety, resulting in a change in physical characteristics as well as a disruption in the normal functioning of the LDL particle. Of course, oxidative modification is not the most important in imparting atherogenic potential to modified LDL. However, in current studies of the mechanisms of atherogenesis, oxidized lipoprotein is often used as a model of modified lipoprotein [104-112].

**Fig. (3):**
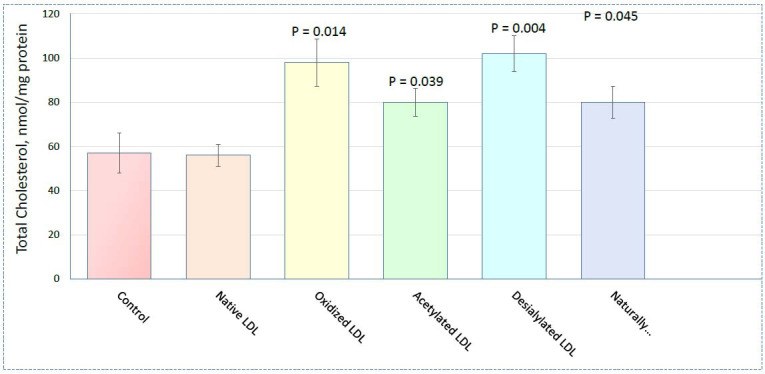
Cholesterol content in cultured macrophages. Adapted from [10]. Total cholesterol in cultured human monocyte-derived macrophages was measured after treatment with different LDL samples. Data represent a mean ±SEM (nmol/mg protein). *P*-value *vs.* control is indicated in parentheses. Differences between control and LDL preparations were identified using the paired sample t-test with 21.0 IBM SPSS Statistics.

However, as shown by recent studies of signaling pathways regulating the accumulation of cholesterol caused by modified lipoproteins, oxidized LDL is a poor model of an atherogenic modified lipoprotein [10]. LDL samples studied in the cited study included native LDL, multiply modified LDL isolated from the blood of atherosclerotic patients (naturally occurring LDL), and LDL modified *in vitro* by acetylation, oxidation, and desialylation. All modified forms of LDL, both naturally occurring LDL and LDL modified *in vitro*, caused a significant and comparable increase in cholesterol content in human monocyte-derived macrophages (Fig. **3**). Signaling pathways have been identified that are regulated in macrophages in response to lipoproteins. All signaling pathways regulated by natural multiply modified LDL were compared with signaling pathways regulated by *in vitro* modified LDL. A complete overlap of signaling pathways that were down-regulated by naturally circulating and *in vitro* desialylated LDL was found. Two other types of atherogenic modifications (acetylation and oxidation) differ sharply from naturally occurring LDL. In the case of acetylated LDL, 29 up-regulated signaling pathways were found that are not regulated by circulating atherogenic multiply modified LDL. Acetylated LDL down-regulates only one signaling pathway matching natural LDL. An even weaker match was found for oxidized LDL which did not down-regulate signaling pathways at all. The only two up-regulated signaling pathways identified for oxidized LDL were matched for *in vitro* oxidized and naturally occurring multiply modified LDL. Thus, oxidized LDL is a poor model of modified lipoprotein, at least for studying gene regulation.

## 10 CLINICAL TRIALS DID NOT SUPPORT THE OXIDATIVE HYPOTHESIS

To become a revolutionary breakthrough in the field of atherosclerosis, the rapid development of basic research generated by the oxidative hypothesis had to effectively culminate in the confirmation of the hypothesis by clinical studies, which were looked at with much optimism. In a 1995 Lancet article with a promising title “Clinical trials of antioxidants in atherosclerosis: are we doing the right thing?”, Dan Steinberg wrote: “antioxidants can slow the atherogenic process in several experimental models, including LDL-receptor-deficient rabbits, cholesterol-fed rabbits, and cholesterol-fed non-human primates” [113]. That is, Steinberg and his followers had great hopes associated with the results of the planned clinical trials.

Optimism persisted until the very beginning of the 2000s. Against the background of the fact that studies *in vitro* and animal models have provided an ever- growing body of direct and indirect evidence for the involvement of low-density lipoprotein oxidation as a key factor in atherogenesis. The fiasco broke out when the results of the clinical trials were summed up. Here is what Steinberg wrote in 2000: “However, two recent, very large, carefully conducted clinical intervention trials using adequate doses of vitamin E demonstrated no effect on a composite end-point of non-fatal infarction, stroke or death from cardiovascular causes” [114]. “The large clinical trials of antioxidants, most of them using vitamin E or beta carotene, have been resoundingly negative. Meta-analysis of the data from these studies (n=≈80,000) shows no benefit at all with regard to cardiovascular outcomes” [48].

It would seem that these facts completely killed the oxidative hypothesis; however, research on oxidized LDL continues. Why?

## 11 WHY IS OXIDIZED LDL STILL A KEY LIPOPROTEIN IN ATHEROGENESIS?

I have no rational answer to this question. The only explanation that comes to mind is due to the huge number of apologists for the oxidative hypothesis who have remained out of work. In accordance with tradition, after receiving negative results of clinical trials, the topic of oxidized LDL was immediately closed and no money was given out for it. A huge army of grantees came up with an “elegant” way out. It was decided to consider the problem of modified LDL completely solved. Now, it is allowed to speak exclusively about the oxidized form of LDL modification; other forms are not even considered. It was decided to completely forget about the failures with clinical studies of antioxidants. Thus, an entire branch of science related to modified LDL was buried. If from the moment the oxidative hypothesis appeared, there was a rapid increase in publications on modified LDL for 15-20 years, then immediately after the publication of data from unsuccessful clinical trials of antioxidants, that is, by the beginning of the 2000s, the growth of publications on this topic slowed down sharply, and for the last 13 years, there has been no growth at all. PubMed database clearly demonstrates this fact.

## 12 ARGUMENTS SUPPORTING AND WEAKENING HYPOTHESES

Summarizing all the above facts, it would be right to increase the clarity of the argument by providing a more structured presentation of evidence confirming or refuting both the oxidative hypothesis and the concept of non-oxidized (multiply modified) LDL. To better understand and clearly delineate each line of evidence, a table is provided below.

Table **2** clearly demonstrates that both oxidized LDL and non-oxidized forms of atherogenic-modified LDL are present in the blood as well as in the vascular wall. The atherogenic potential of oxidized and non-oxidized modified LDL has been shown both *in vitro* and *in vivo* in animal models [48, 115].

However, the key role of oxidized LDL in atherogenesis is questionable since oxidized LDL has not been isolated from human blood, while non-oxidized forms of modified LDL have not only been isolated by various methods but have also been intensively and extensively studied in detail. Autoantibodies against modified LDL found in human blood are not specific for oxidized LDL but rather are antigenized by non-oxidized modifications of LDL. Circulating immune complexes (CICs) obtained from the blood of athero- sclerotic patients do not contain exclusively oxidized LDL but rather include multiple-modified LDL particles [116]. In the cited work, when comparing LDL from CIC with native and desialylated LDL isolated from the same blood and separated by lectin chromatography on RCA120-agarose, it was found that “the levels of lipid-oxidation products, thiobarbituric acid-reactive substances and hydroperoxides, were similar in all lipoprotein preparations. However, desialylated LDL and CIC-LDL had an elevated oxysterol content.” In addition, “the mean diameters of native, desialylated and CIC-LDL accounted for 24.0, 21.3 and 19.5 nm, respectively. Like desialylated LDL, CIC-LDL displayed higher electrophoretic mobility compared with that of native LDL. Thus, LDL obtained from circulation immune complexes appear to be a multiple-modified lipoprotein.”

A comparison of the signaling pathways involved in the accumulation of intracellular cholesterol caused by atherogenic LDL isolated from the blood of atherosclerotic patients and various forms of *in vitro* modified LDL showed that the most similar to naturally occurring modified LDL is LDL desialylated *in vitro*. Oxidized LDL was dramatically different from the modified LDL circulating in the blood of patients.

As for clinical data, they did not confirm the key role of oxidized LDL in atherogenesis. Diagnostic tests designed to assess the amount of oxidized LDL in the blood of patients are not specific for oxidized LDL but may overlap with other forms of modified LDL [2, 117]. Clinical trials of antioxidants that theoretically should inhibit LDL oxidation have been resoundingly negative. On the other hand, neither positive nor negative clinical data revealing the role of non-oxidized forms of modified LDL in atherogenesis have yet been obtained. This is a weak side of the system of evidence of the participation of non-oxidized forms of modified LDL in the occurrence and development of atherosclerosis, but over time, such data will be obtained if the attitude towards non-oxidized atherogenic LDL changes and its study takes its rightful place in the current study of the mechanisms of atherosclerosis.

Thus, oxidized LDL is present in the blood of patients but has not been isolated, probably due to its low proportion in the total amount of circulating lipoprotein particles. Oxidized LDL is certainly present in the vascular wall, which is natural since aging particles accumulate there. One could assume that the oxidative modification of LDL does not occur in the blood but rather in the vascular wall, where the atherogenic potential of oxidized LDL is realized. However, firstly, this cannot be shown directly, and secondly, both in the blood and the vascular wall, non-oxidized forms of modified LDL have been found and studied in detail, exhibiting a high atherogenic potential.

So, it is not the existence of oxidized LDL that is being questioned, but its unproven mythical key role in atherogenesis. Science is not a religion and pure faith is not enough for scientists; but they need evidence, which is still lacking.

## CONCLUSION

Atherogenic modified LDL is an important and interesting topic. The development of this line still promises many discoveries related to the diagnosis, prevention and treatment of atherosclerotic diseases. However, due to a bizarre set of circumstances, the topic received a very one-sided development, focusing exclusively on the mythical oxidized LDL. This greatly complicates the study of actual forms of atherogenic modification of lipoproteins. To productively study the role of modified LDL in atherosclerosis, it is necessary to reconsider the unworkable paradigm. We must abandon the myth! The outdated and unrealistic oxidative hypothesis is leading astray. It does not allow studying the true mechanisms of atherogenesis and also hinders the development of approaches to the diagnosis and treatment of atherosclerotic diseases.

## Figures and Tables

**Table 1:** Changes of characteristics of Real Modified LDL Isolated from Human Blood related to native LDL.

**Characteristic**	**Desialylated LDL**	**LDL(-)**	**Small Dense LDL**
Sialic acid	↓ [62, 84]	↓ [81, 85]	↓ [83]
Cholesteryl esters	↓ [82]	↓ [69]	↓ [86, 87]
Phospholipids	↓ [82]	↓ [69]	↓ [86, 87]
Antioxidants	↓ [82, 89]	↓ [69]	↓ [88, 99]
Protein/lipid ratio	↑ [82]	↑ [69]	↑ [91, 86]
Amino group modification	↑ [82]	no data	↑ [90]
Size	↓ [82, 89]	↓ [67]	↓ [92, 93]
Density	↑ [82, 89]	no data	↑ [86]
(-) Charge	↑ [82]	↑ [67]	↑ [92]
Atherogenicity (intracellular lipid accumulation)	↑ [62, 84]	↑ [67]	↑ [92, 94]
Self-association	↑ [95, 96]	↑ [97]	↑ [96]
Oxidizability (susceptibility to oxidation)	↑ [94]	↑ [69]	↑ [88, 99]

**Note: ** Adapted from [100]. The table presents data obtained by three different groups studying desialylated LDL (Tertov *et al.*, Russia), LDL(-) (Avogaro *et al.*, Italy) and small dense LDL (Krauss *et al.*, USA). LDL was isolated from the blood of atherosclerotic patients. Desialylated LDL subfunction was separated by lectin chromatography (affinity chromatography on *Ricinus communis* agglutinin-agarose) as described elsewhere [82]. LDL(-) subfunction was separated by ion exchange chromatography on DEAE Sepharose or by ion exchange high-performance liquid chromatography [67, 68]. Small dense LDL subfraction was separated by density gradient centrifugation and confirmed by gradient gel electrophoresis [99].

**Table 2:** Strengths and weaknesses of each line of evidence.

**Argument**	**Oxidized LDL**	**Non-oxidized LDL**
*In vitro* atherogenicity*	proven	proven
*In vivo* atherogenicity**	proven	proven
Presence in human blood	proven	proven
Presence in artery wall	proven	proven
Isolated from human blood	no	yes
Autoantibodies	low affinity	high affinity
Contained in circulating immune complexes	not isolated	isolated and studied in-depth
Model for mechanistic study, including signaling	bad	good
Diagnostic test	inadequate	not available
Support by clinical data	unsuccessful	not studied

**Note: ** *, Ability to cause accumulation of intracellular lipids.

**, Ability to induce atherosclerotic-like lesions in animal models.

## LIST OF ABBREVIATIONS

CICs: Circulating Immune Complexes
LDL: Low-density Lipoprotein
LDL(-): Electronegative Low-density Lipoprotein
MDA: Malondialdehyde
sdLDL: Small Dense Low-density Lipoprotein
